# Supplementary material for: Vitamin D levels of pregnant immigrant women and developmental disorders of language, learning and coordination in offspring
Source: PLoS One. 2024 Feb 29;19(2):e0299808. doi: 10.1371/journal.pone.0299808 (PMC10903893; doi:10.1371/journal.pone.0299808)
Supplement: S1 File — (DOCX) [file pone.0299808.s001.docx]

**S1 Table 1: Relationship between 1) covariates and maternal serum vitamin D among immigrant mothers to controls, and 2) covariates and language, coordination, scholastic and mixed developmental disorder diagnosis in immigrant case and control subjects.**

|  | **Covariates and continuous* vitamin D among immigrant mothers to controls** | | **Covariates and outcome diagnoses** | | |
| --- | --- | --- | --- | --- | --- |
| **Categorical variables** | **Mean (SD)**  **Nmol/l** | **P-value** | **Immigrant mothers to cases (N=443)**  **n (%)** | **Immigrant mothers to controls (N=443)**  **n (%)** | **P-value** |
| **Maternal smoking** |  | 0.29 |  |  | 0.15 |
| No | 25.4 (15.5) |  | 416 (99.0) | 416 (97.9) |  |
| Yes | 32.6 (22.9) |  | 4 (1.0) | 9 (2.1) |  |
| **Previous births** |  | 0.03 |  |  | <0.001 |
| 0 | 27.8 (17.1) |  | 178 (40.5) | 128 (29.1) |  |
| ≥ 1 | 24.4 (14.8) |  | 262 (59.5) | 312 (70.9) |  |
| **Maternal psychiatric history**^a^ |  | 0.67 |  |  | 0.92 |
| No | 25.0 (15.0) |  | 392 (88.5) | 391 (88.3) |  |
| Yes | 27.9 (19.0) |  | 51 (11.5) | 52 (11.7) |  |
| **Paternal psychiatric history^b^** |  | 0.94 |  |  | 0.04 |
| No | 25.3 (15.4) |  | 375 (87.0) | 395 (91.0 |  |
| Yes | 24.6 (15.4) |  | 56 (13.0) | 39 (9.0) |  |
| **Maternal SES** |  | <0.001 |  |  | 0.22 |
| Upper white collar | 25.9 (14.6) |  | 13 (3.9) | 31 (8.9) |  |
| Lower white collar | 31.9 (20.7) |  | 46 (13.8) | 36 (10.4) |  |
| Blue collar | 27.6 (11.5) |  | 52 (15.6) | 52 (15.0) |  |
| Others | 22.9 (13.5) |  | 223 (66.8) | 228 (65.7) |  |
| **Maternal substance abuse^c^** |  | 0.62 |  |  | 0.99 |
| No | 25.4 (15.5) |  | 443 (100.0) | 442 (99.8) |  |
| Yes | 28.5 |  | 0 (0.0) | 1 (0.2) |  |
| **Gestational diabetes** |  | 0.93 |  |  | 0.02 |
| No | 25.5 (15.9) |  | 409 (92.3) | 388 (87.6) |  |
| Yes | 24.1 (12.5) |  | 34 (7.7) | 55 (12.4) |  |
| **Weight for gestational age** |  | 0.59 |  |  | 0.22 |
| <-2 SD | 25.6 (14.4) |  | 35 (8.0) | 27 (6.2) |  |
| -2 SD to +2 SD | 25.3 (15.5) |  | 400 (91.3) | 403 (92.0) |  |
| >+2 SD | 31.2 (22.0) |  | 3 (0.7) | 8 (1.8) |  |
| **Gestational age** |  | 0.99 |  |  | 0.05 |
| <37 weeks | 27.6 (20.7) |  | 30 (6.8) | 16 (3.7) |  |
| ≥37 weeks | 25.3 (15.4) |  | 409 (93.2) | 422 (96.4) |  |
| **Season of blood draw** |  | <0.001 |  |  | 0.34 |
| Spring | 22.9 (12.0) |  | 138 (31.2) | 124 (28.1) |  |
| Summer | 31.4 (19.4) |  | 115 (26.0) | 102 (23.1) |  |
| Autumn | 24.5 (14.7) |  | 93 (21.0) | 104 (23.5) |  |
| Winter | 23.5 (14.5) |  | 97 (21.9) | 112 (25.3) |  |
| **Apgar Score** |  | 0.79 |  |  | 0.11 |
| 0–6 | 24.6 (11.4) |  | 41 (9.3) | 29 (6.6) |  |
| 7–10 | 25.4 (15.8) |  | 398 (90.7) | 409 (93.4) |  |
| **Continous variables** | **Parameter estimate (SE)** | **p** | **Mean (SD)** | **Mean (SD)** | **p** |
| Maternal age (years) | 0.01 (0.005) | 0.03 | 27.9 (5.6) | 28.5 (5.5) | 0.09 |
| Gestational week of blood draw | -0.007 (0.005) | 0.14 | 12.8 (5.4) | 12.5 (5.4) | 0.38 |

*Analysis performed with log-transformed vitamin D levels due to a skewed distribution.

^a^ ICD-8 (291-308), ICD-9 (291-316) or ICD-10 (F10-99, excluding maternal substance abuse diagnosis, ^b^ ICD-8 (291-308), ICD-9 (291-316) or ICD-10 (F10-99); ^c^ ICD-8 (291, 303, 304), ICD-9 (291, 292, 303,304,305) or ICD-10 (F10-19). Abbreviations: SES, socioeconomic status. SD, standard deviation. SE, standard error.

Maternal substance abuse comparison among controls not applicable due to no controls in group “yes”. Missing data: Maternal smoking 18 controls, 23 cases. Previous births 3 controls, 3 cases. Paternal psychiatric history 9 controls, 12 cases. Maternal SES 96 controls, 109 cases. Weight for gestational age, 5 controls, 5 cases. Gestational age 5 controls, 4 cases. Apgar score 5 controls, 4 cases. Season of blood draw 1 control, 0 cases. Gestational week of blood draw 6 controls, 4 cases.

**S1 Table 2: Relationship between 1) covariates and maternal serum vitamin D Finnish mothers to controls, and 2) covariates and language, coordination, scholastic and mixed developmental disorder diagnosis in case and control subjects.**

|  | **Covariates and continuous* vitamin D among Finnish mothers to controls** | | **Covariates and outcome diagnoses** | | |
| --- | --- | --- | --- | --- | --- |
| **Categorical variables** | **Mean (SD)**  **Nmol/l** | **P-value** | **Immigrant mothers to cases (N=542)**  **n (%)** | **Finnish mothers to controls (N=542)**  **n (%)** | **P-value** |
| **Maternal smoking** |  | 0.40 |  |  | <0.001 |
| No | 42.4 (19.1) |  | 501 (92.4) | 445 (82.1) |  |
| Yes | 41.1 (19.5) |  | 8 (1.5) | 79 (14.6) |  |
| **Previous births** |  | 0.67 |  |  | 0.85 |
| 0 | 42.7 (19.1) |  | 213 (39.3) | 218 (40.2) |  |
| ≥ 1 | 41.9 (19.1) |  | 322 (59.4) | 323 (59.6) |  |
| **Maternal psychiatric history**^a^ |  | 0.005 |  |  | 0.21 |
| No | 42.97 (18.7) |  | 479 (88.4) | 465 (85.8) |  |
| Yes | 37.97 (21.2) |  | 63 (11.6) | 77 (14.2) |  |
| **Paternal psychiatric history^b^** |  | 0.04 |  |  | 1.00 |
| No | 42.6 (19.2) |  | 461 (85.0) | 468 (86.3) |  |
| Yes | 38.0 (17.7) |  | 66 (12.2) | 67 (12.4) |  |
| **Maternal SES** |  | 0.57 |  |  | <0.001 |
| Upper white collar | 43.9 (19.6) |  | 14 (2.6) | 102 (18.8) |  |
| Lower white collar | 41.5 (18.4) |  | 54 (10.0) | 231 (42.6) |  |
| Blue collar | 41.4 (19.3) |  | 64 (11.8) | 86 (15.9) |  |
| Others | 43.2 (19.7) |  | 268 (49.4) | 86 (15.9) |  |
| **Maternal substance abuse^c^** |  | 0.50 |  |  | 0.97 |
| No | 42.3 (19.1) |  | 542 (100.0) | 534 (98.5) |  |
| Yes | 39.4 (24.5) |  | 0 (0.0) | 8 (1.5) |  |
| **Gestational diabetes** |  | 0.39 |  |  | 1.00 |
| No | 42.5 (19.4) |  | 493 (91.0) | 493 (91.0) |  |
| Yes | 39.5 (16.5) |  | 49 (9.0) | 49 (9.0) |  |
| **Weight for gestational age** |  | 0.01 |  |  | <0.001 |
| <-2 SD | 29.5 (11.7) |  | 42 (7.7) | 14 (2.6) |  |
| -2 SD to +2 SD | 42.8 (19.3) |  | 487 (89.9) | 513 (94.6) |  |
| >+2 SD | 37.1 (11.1) |  | 3 (0.6) | 14 (2.6) |  |
| **Gestational age** |  | 0.10 |  |  | 0.03 |
| <37 weeks | 35.9 (20.4) |  | 30 (5.5) | 15 (2.8) |  |
| ≥37 weeks | 42.5 (19.1) |  | 504 (93.0) | 526 (97.0) |  |
| **Season of blood draw** |  | <0.001 |  |  | 0.14 |
| Spring | 34.7 (14.6) |  | 159 (29.3) | 128 (23.6) |  |
| Summer | 57.2 (21.8) |  | 135 (24.9) | 133 (24.5) |  |
| Autumn | 41.9 (14.8) |  | 120 (22.1) | 120 (22.1) |  |
| Winter | 35.5 (14.8) |  | 128 (23.6) | 140 (25.8) |  |
| **Apgar Score** |  | 0.02 |  |  | 0.004 |
| 0–6 | 46.8 (12.9) |  | 47 (8.7) | 23 (4.2) |  |
| 7–10 | 42.1 (19.3) |  | 488 (90.0) | 519 (95.8) |  |
| **Continous variables** | **Parameter estimate (SE)** | **p** | **Mean (SD)** | **Mean (SD)** | **p** |
| Maternal age (years) | 0.007 (0.003) | 0.03 | 28.0 (5.4) | 29.6 (5.4) | <0.001 |
| Gestational week of blood draw | 0.005 (0.007) | 0.47 | 12.7 (5.6) | 9.7 (2.6) | <0.001 |

*Analysis performed with log-transformed vitamin D levels due to a skewed distribution.

^a^ ICD-8 (291-308), ICD-9 (291-316) or ICD-10 (F10-99, excluding maternal substance abuse diagnosis, ^b^ ICD-8 (291-308), ICD-9 (291-316) or ICD-10 (F10-99); ^c^ ICD-8 (291, 303, 304), ICD-9 (291, 292, 303,304,305) or ICD-10 (F10-19). Abbreviations: SES, socioeconomic status. SD, standard deviation. SE, standard error. Missing data: Maternal smoking 18 controls, 33 cases. Previous births 1 control, 7 cases. Paternal psychiatric history 7 controls, 15 cases. Maternal SES 37 controls, 142 cases. Weight for gestational age, 1 control, 10 cases. Gestational age 0 controls, 7 cases. Apgar score 0 controls, 7 cases. Season of blood draw 21 controls, 0 cases. Gestational week of blood draw 22 controls, 8 cases.
