# Supplementary material for: Vitamin D levels of pregnant immigrant women and developmental disorders of language, learning and coordination in offspring
Source: PLoS One. 2024 Feb 29;19(2):e0299808. doi: 10.1371/journal.pone.0299808 (PMC10903893; doi:10.1371/journal.pone.0299808)
Supplement: S1 Appendix — (DOCX) [file pone.0299808.s002.docx]

**S2 Appendix. Birth countries of immigrant mothers to cases and controls.**

*Sub Saharan Africa*

Angola

South Africa

Ethiopia

Eritrea

Gambia

Ghana

Guinea

Camerun

Kap Verde

Kenya

Kongo

Liberia

Namibia

Nigeria

Ivory Coast

Zambia

Sierra Leone

Somalia

Tanzania

Togo

Uganda

*North Africa and Middle East*

Egypt

Yemen

Morocco

Syria

*South and Central America*

Bolivia

El Salvador

Honduras

Nicaragua

*Asia*

Afganistan

Bangladesh

Philippines

Indonesia

India

Iraq

Cambodzha

Myanmar

Nepal

Pakistan

Vietnam

Uzbekistan
