# Supplementary material for: Vitamin D levels of pregnant immigrant women and developmental disorders of language, learning and coordination in offspring
Source: PLoS One. 2024 Feb 29;19(2):e0299808. doi: 10.1371/journal.pone.0299808 (PMC10903893; doi:10.1371/journal.pone.0299808)
Supplement: S1 Table — (DOCX) [file pone.0299808.s003.docx]

| Characteristic | Immigrant cases with an immigrant control  N=443  n (%) | Immigrant cases without an immigrant control  N=109  n (%) | P-value* |
| --- | --- | --- | --- |
| Sex (male) | 314 (70.9) | 81 (74.3) | 0.55 |
| Mother’s birth continent  Asia  Sub Saharan Africa  North Africa and Middle East  Latin or South America | 224 (50.6)  187 (42.2)  27 (6.1)  5 (1.1) | 62 (56.9)  38 (34.9)  7 (6.4)  2 (1.8) | 0.53 |
| Diagnosed developmental disorder(s)    Language/speech disorder  Scholastic disorder  Coordination disorder  Mixed developmental disorder | 322 (72.7)  60 (13.5)  40 (9.0)  135 (35.0) | 84 (77.0)  12 (11.0)  11 (10.1)  29 (26.6) | 0.40  0.53  0.71  0.11 |

S3 Table. Descriptive characteristics of immigrant subjects with and without immigrant controls.

*Chi-square or Fisher’s exact test
