# Supplementary material for: Vitamin D levels of pregnant immigrant women and developmental disorders of language, learning and coordination in offspring
Source: PLoS One. 2024 Feb 29;19(2):e0299808. doi: 10.1371/journal.pone.0299808 (PMC10903893; doi:10.1371/journal.pone.0299808)
Supplement: S2 Table — (DOCX) [file pone.0299808.s004.docx]

| Year of blood draw | Immigrant mothers of cases^a^ | | Immigrant mothers of controls^b^ | | Finnish mothers of controls^c^ | |
| --- | --- | --- | --- | --- | --- | --- |
|  | N | Mean (SD) | N | Mean (SD) | N | Mean (SD) |
| 1995 | 13 | 22.6 (12.3) | 9 | 25.5 (11.2) | 18 | 47.1 (20.2) |
| 1996 | 27 | 28.5 (15.9) | 34 | 26.2 (18.6) | 29 | 40.6 (17.3) |
| 1997 | 42 | 25.7 (19.5) | 27 | 30.7 (20.5) | 41 | 39.0 (18.6) |
| 1998 | 43 | 25.5 (13.9) | 42 | 22.3 (15.7) | 39 | 37.1 (17.4) |
| 1999 | 49 | 25.7 (15.8) | 40 | 23.3 (12.2) | 46 | 37.8 (19.2) |
| 2000 | 61 | 25.7 (16.3) | 49 | 21.2 (14.3) | 67 | 42.5 (19.3) |
| 2001 | 63 | 24.7 (15.2) | 63 | 24.6 (14.8) | 61 | 40.2 (20.2) |
| 2002 | 47 | 20.5 (11.0) | 46 | 26.3 (15.2) | 52 | 39.7 (18.7) |
| 2003 | 51 | 24.3 (11.5) | 31 | 25.8 (14.4) | 50 | 47.1 (23.4) |
| 2004 | 72 | 26.2 (13.0) | 46 | 30.2 (18.2) | 73 | 46.7 (16.1) |
| 2005 | 56 | 24.1 (12.0) | 45 | 26.6 (11.9) | 54 | 44.3 (18.5) |
| 2006 | 18 | 26.2 (14.3) | 11 | 21.1 (14.0) | 12 | 47.6 (18.4) |

**S4 Table****. Yearly means of maternal vitamin D in each group of cases and controls.** A dashed line marks the vitamin D supplementation recommendation and food fortification start in Finland in 2003.

^a^T-test for mean vitamin D levels 1995-2002 vs 2003-2006 among immigrant mothers of cases, p= 0.22

^b^T-test for mean vitamin D levels 1995-2002 vs 2003-2006 among immigrant mothers of controls, p= 0.01

^c^T-test for mean vitamin D levels 1995-2002 vs 2003-2006 among Finnish mothers of controls, p= <0.001
