# Supplementary material for: Vitamin D levels of pregnant immigrant women and developmental disorders of language, learning and coordination in offspring
Source: PLoS One. 2024 Feb 29;19(2):e0299808. doi: 10.1371/journal.pone.0299808 (PMC10903893; doi:10.1371/journal.pone.0299808)
Supplement: Attachment — Submitted filename: Response to reviewers.docx [file pone.0299808.s005.docx]

**Response letter**

A) **Editorial comments**

When submitting your revision, we need you to address these additional requirements.

1. Please ensure that your manuscript meets PLOS ONE's style requirements, including those for file naming. The PLOS ONE style templates can be found at

https://journals.plos.org/plosone/s/file?id=wjVg/PLOSOne_formatting_sample_main_body.pdf and

<https://journals.plos.org/plosone/s/file?id=ba62/PLOSOne_formatting_sample_title_authors_affiliations.pdf>

***Response:*** *we have checked the formatting requirements and modified the manuscript accordingly.*

2. We note that the grant information you provided in the ‘Funding Information’ and ‘Financial Disclosure’ sections do not match.

When you resubmit, please ensure that you provide the correct grant numbers for the awards you received for your study in the ‘Funding Information’ section.

***Response:*** *the grant information has been checked and corrected accordingly (Academy of Finland and Research Council of Finland are the same organization, this has been clarified now).*

Thank you for stating the following financial disclosure:

"This research was supported supported by the INVEST Research Flagship Centre/Research Council of Finland (decision number: 345546)."

Please state what role the funders took in the study. If the funders had no role, please state: "The funders had no role in study design, data collection and analysis, decision to publish, or preparation of the manuscript."

If this statement is not correct you must amend it as needed.

Please include this amended Role of Funder statement in your cover letter; we will change the online submission form on your behalf.

***Response:*** *the role of the funder has been added to the cover letter.*

3. We note that you have indicated that data from this study are available upon request. PLOS only allows data to be available upon request if there are legal or ethical restrictions on sharing data publicly. For more information on unacceptable data access restrictions, please see http://journals.plos.org/plosone/s/data-availability#loc-unacceptable-data-access-restrictions.

In your revised cover letter, please address the following prompts:

a) If there are ethical or legal restrictions on sharing a de-identified data set, please explain them in detail (e.g., data contain potentially sensitive information, data are owned by a third-party organization, etc.) and who has imposed them (e.g., an ethics committee). Please also provide contact information for a data access committee, ethics committee, or other institutional body to which data requests may be sent.

b) If there are no restrictions, please upload the minimal anonymized data set necessary to replicate your study findings as either Supporting Information files or to a stable, public repository and provide us with the relevant URLs, DOIs, or accession numbers. For a list of acceptable repositories, please see http://journals.plos.org/plosone/s/data-availability#loc-recommended-repositories.

We will update your Data Availability statement on your behalf to reflect the information you provide.

***Response:*** *the legal restrictions that prohibit data sharing have been precised in the cover letter.*

4. We note that you have included the phrase “data not shown” in your manuscript. Unfortunately, this does not meet our data sharing requirements. PLOS does not permit references to inaccessible data. We require that authors provide all relevant data within the paper, Supporting Information files, or in an acceptable, public repository. Please add a citation to support this phrase or upload the data that corresponds with these findings to a stable repository (such as Figshare or Dryad) and provide and URLs, DOIs, or accession numbers that may be used to access these data. Or, if the data are not a core part of the research being presented in your study, we ask that you remove the phrase that refers to these data.

***Response:*** *We have added S3 Table with the relevant supporting data and removed the phrase “data not shown”.*

5. Please review your reference list to ensure that it is complete and correct. If you have cited papers that have been retracted, please include the rationale for doing so in the manuscript text, or remove these references and replace them with relevant current references. Any changes to the reference list should be mentioned in the rebuttal letter that accompanies your revised manuscript. If you need to cite a retracted article, indicate the article’s retracted status in the References list and also include a citation and full reference for the retraction notice.

***Response:*** *The reference list has been checked and is correct. No changes were made to the references.*

B) **Reviewers' comments**

Reviewer #1:

1) what the criteria of low or medium human development index (HDI) line 150

***Response:*** *Thank you for pointing this out. We have added information on the HDI categorization to the manuscript (page 6, lines 173-177). The reference provided (ref 14) also offers extensive information on the categorization.*

2) line 158 Coordination disorders are characterized by impaired fine and gross motor coordination development no surgical or accidents matters

***Response:*** *We have added this precision to page 6, line 181.*

3) line 200 25(OH)D vitamin D or 1, 25 Di (OH) D

***Response:*** *This line was revised accordingly (line 228).*

4) vitamin D have role in immunity do you have any results about immunity or disease resistance

***Response:*** *Thank you for the suggestion. Unfortunately, we do not have any data on immunological markers for this sample.*

Reviewer #2: Prenatal vitamin D levels of immigrant mothers and language, scholastic, coordination and mixed developmental disorders in offspring

Vitamin D deficiency causes health issues not only in children as well in young and old age livings both in human and animals. Prenatal vitamin D levels of immigrant mothers and language, scholastic, coordination and mixed developmental disorders in offspring is an interesting study with respect to immigrant population in Finland. I will suggest few changes in the manuscript before publication. These changes are following:

5) On line # 84: write full spellings of ADHD as used first time in the text.

***Response:*** *“attention deficit hyperactivity disorder (ADHD)” has been added to the text, line 103.*

6) On line # 229: Can authors add data of % age of different countries rather than immigrant’s continents having vit D deficiency used in the present study samples?

***Response:*** *We did not fully understand this comment. What is meant by “Age of different countries?” However, the S2 Appendix provides a list of all the included immigrant mothers’ birth countries.*

7) was there any deficiency of Ca, P etc in immigrants mothers related with the deficiency of vit D?

***Response:*** *Thank you for the question. Unfortunately, nor calcium or phosphate were measured from the study subjects’ blood samples.*

8) is there any relationship between the year of migration of immigrant’s mothers, present study and Vit D deficiency?

***Response:*** *Thank you for the excellent question. We checked this: the time from immigration date to blood draw was not associated with the vitamin D levels among immigrant mothers. We added this to the results, page 10, lines 284-288.*

9) Need to improve table #02 for readers.

***Response:*** *Thank you for the suggestion. The table has been modified a little bit to be more reader-friendly, but the journal’s requirements prohibit the use of e.g., extra spacing, so the changes were minor. Please suggest more specific revisions to the table if needed.*

10) Reviewer #3: I do not like the title of the paper. It needs to be made more precise. Otherwise, the paper is a very good attempt. Methodology is sound. Discussion is extremely comprehensive. Methodology is well detailed.

***Response:*** *Thank you for the positive feedback. We changed the title to “**Vitamin D levels of pregnant immigrant women and developmental disorders of language, learning and coordination in offspring” We are also open to other suggestions for the title.*

11) Reviewer #4: Congrats to the authors on their hard work. It is an original study—the introduction, discussion, and conclusion were well-written and robust statistical analysis performed. I would advise the author to consider enhancing the visual cues by adding more graphics, not just tables.

***Response:*** *Thank you for the positive feedback! We have added a figure on the vitamin D level changes before and after the food fortification and recommendation change in Finland in 2003 (Fig 1).*
